# Evaluation of Plasmodium falciparum gametocyte detection in different patient material

**DOI:** 10.1186/1475-2875-12-438

**Published:** 2013-12-04

**Authors:** Katharina Kast, Nicole Berens-Riha, Ahmed Zeynudin, Nuredin Abduselam, Teferi Eshetu, Thomas Löscher, Andreas Wieser, Jonathan Shock, Michael Pritsch

**Affiliations:** 1 Division of Infectious Diseases and Tropical Medicine, Medical Centre of the University of Munich (LMU), Leopoldstrasse 5, 80802 Munich, Germany; 2 German Centre for Infection Research (DZIF), partner site Munich, Leopoldstrasse 5, 80802 München (Munich), Germany; 3 Max von Pettenkofer-Institute of Hygiene and Medical Microbiology, Leopoldstrasse 5, 80802 Munich, Germany; 4 Department of Microbiology, Parasitology and Immunology, Jimma University, Jimma, Ethiopia; 5 The Laboratory for Quantum Gravity & Strings, Department of Mathematics and Applied Mathematics, University of Cape Town, Cape Town, South Africa

## Abstract

**Background:**

For future eradication strategies of malaria it is important to control the transmission of gametocytes from humans to the anopheline vector which causes the spread of the disease. Sensitive, non-invasive methods to detect gametocytes under field conditions can play a role in monitoring transmission potential.

**Methods:**

Microscopically *Plasmodium falciparum*-positive patients from Jimma, Ethiopia donated finger-prick blood, venous blood, saliva, oral mucosa and urine samples that were spotted on filter paper or swabs. All samples were taken and stored under equal, standardized conditions. RNA was extracted from the filter paper and detected by real-time QT-NASBA. Pfs16-mRNA and Pfs25-mRNA were measured with a time to positivity to detect gametocyte specific mRNA in different gametocyte stages. They were compared to 18S-rRNA, which is expressed in all parasite stages. Results were quantified via a known dilution series of artificial RNA copies.

**Results:**

Ninety-six samples of 16 uncomplicated malaria patients were investigated. 10 (66.7%) of the slides showed gametocyte densities between 0.3-2.9 gametocytes/μl. For all RNA-targets, molecular detection in blood samples was most sensitive; finger-prick sampling required significantly smaller amounts of blood than venous blood collection. Detection of asexual 18S-rRNA in saliva and urine showed sensitivities of 80 and 67%, respectively. Non-invasive methods to count gametocytes proved insensitive. Pfs16-mRNA was detectable in 20% of urine samples, sensitivities for other materials were lower. Pfs25-mRNA was not detectable in any sample.

**Conclusions:**

The sensitivity of non-invasively collected material such as urine, saliva or mucosa seems unsuitable for the detection of gametocyte-specific mRNA. Sensitivity in asymptomatic carriers might be generally even lower. Finger-prick testing revealed the highest absolute count of RNA copies per μL, especially for Pfs25-mRNA copies. The method proved to be the most effective and should preferably be applied in future transmission control and eradication plans. A rapid test for gametocyte targets would simplify efforts.

## Background

The presence of mature *Plasmodium falciparum* sexually committed parasites, so-called stage V gametocytes, is necessary for transmission from humans to anopheline vectors, while the asexual stages are responsible for the clinical symptoms of the disease.

Malaria remains one of the world’s leading health problems. In 2010, about 216 million cases of malaria and an estimated 665,000 deaths occurred. Approximately half of the world’s population is at risk of malaria [1]. Studies investigating the infectious reservoir were for a long time neglected, but recognition of their relevance has increased in the recent years as eradication of malaria is on the agenda of many countries [2].

It was previously shown that gametocyte densities below the detection limit of microscopy can still lead to mosquito infection [3-6]. To bridge this gap and to quantify the infectious reservoir, modern molecular techniques were introduced [7,8]. In this study, quantitative nucleic acid sequence based amplification (QT-NASBA) was used, which offers a limit of detection (LOD) down to 0.02 gametocytes per μL [9].

An obstacle for large-scale epidemiological investigations in the context of malaria elimination and eradication is the necessity to repeatedly take blood samples from clinically healthy individuals by finger prick or venepuncture. Apart from ethical difficulties concerning invasive methods in potentially healthy carriers, risks of infection for the examiners via blood contact as well as cultural taboos impede such studies. Repeated blood sampling is especially problematic in the population groups that are affected frequently: infants, young children and pregnant women.

It was shown in previous studies that the DNA of asexual parasites of *P. falciparum* can be detected by PCR from urine and saliva samples. Results from saliva and urine had a significant or at least positive correlation with microscopy counts [10,11]. To differentiate between sexual and asexual forms in malaria, RNA amplification is necessary. RNA detection in tissue and saliva is becoming established i.e. for mycobacterioses to evaluate therapy success [12-14].

In this small pilot study, the detection of gametocyte specific mRNA from microscopically *P. falciparum*-positive patients was investigated in urine and saliva samples as well as mucosa swabs. Results were compared with the detection of 18S-rRNA, which is expressed in all parasite stages. Furthermore, the quantity of gametocyte specific mRNA from finger-prick samples was compared with venepuncture samples.

## Methods

### Study area and patients

The study was conducted in the Jimma Zone in Oromiya regional state, around its capital in the hilly area at around 1650 m above sea level. The city of Jimma is located 255 km southwest of the Ethiopian capital, Addis Ababa. The area is malaria endemic with a low to moderate transmission. The predominant species in most areas is *P. falciparum*, followed by *Plasmodium vivax*. Most cases occur from September to December after the main rainy season from June to August. Recruitment of study patients took place in September 2012. The study site was K'Och'e Health Centre, 30 km northwest of Jimma town. The study was implemented during the routine diagnostic practices of the health centre. Blood slides were collected from all patients with fever. Only *P. falciparum* mono-infection was considered for the study. After microscopic confirmation, patients were informed about the study and included if consent was given.

Inclusion criteria were age above 14 years, uncomplicated mono-infection with *P. falciparum* malaria, parasite densities up to 100,000/μL and informed consent by the patient or the legal representative when appropriate. Exclusion criteria were any anti-malarial treatment within the previous three weeks (anti-malarials such as chloroquine, artemether-lumefantrine, quinine and others as well as antibiotics with anti-malarial effects such as doxycycline, clindamycine), mixed plasmodial infection, danger signs (inability to drink, repeated vomiting, recent history of convulsions, lethargy or unconsciousness) and signs of severe malaria as defined by the WHO [15] as well as pregnancy or lactation.

### Ethical considerations

Each patient (or parent/guardian) received oral and written information about the study and signed an informed consent form. Ethical approval was obtained from the Ethical Committee of the Faculty of Medicine of the Ludwig-Maximilian-University (LMU) in Germany and from the Ethical Board of Jimma University, the Ethiopian Federal Ministry of Science and Technology, in Ethiopia.

### Sample collection and handling

Blood slides were prepared at the initial presentation for all patients with fever or history of fever within 24 hours, during routine examination at the study centre. Sampling and preparation of thin and thick smears were performed by physicians or laboratory technicians who had been trained and instructed by the investigators at the beginning of the study. For determination of parasitaemia and gametocytaemia, thick blood smears of 10 μL blood were prepared according to standard procedures. Slides were stained with 10% Giemsa for 20 min and independently read by two experienced microscopists. Asexual parasites were counted on thick blood films against 200 white blood cells (WBCs). The parasite density was calculated assuming an average WBC count of 8,000/μL. Slides were declared negative if no parasites were seen in 200 fields of the thick film.

After recruitment, different samples were taken before treatment was initialized. Venous blood was drawn from the left bend of the elbow if right handed, and otherwise *vice versa*. A finger-prick examination was then performed. The patients were asked to donate urine and saliva samples. Furthermore, two independent mucosal swabs were taken. One swab was dried and frozen directly. The other was spread on filter paper (Standard-Whatman Cellulose Chromatography paper 3MM, GE Healthcare, Fairfield, CT, USA) immediately after taking the sample. Three lots of 100 μL of saliva, urine and venous blood, respectively, as well as 25 μL blood from finger prick were spotted on filter papers. Those were dried for three hours at room temperature. After drying, all samples were individually sealed in labelled plastic bags and stored in a freezer at -30°C. Samples were transported to Germany on dry ice and processed consecutively. All samples were analysed within one month. During this process, filter papers were stored at -20°C to avoid decay.

### RNA extraction

For analysis, the samples containing 100 μL of venous blood, 100 μL of urine, 100 μL of saliva, 25 μL of finger-prick blood and the mucosal swab spread on filter paper were cut out of the filter paper with heat sterilized scissors maintaining a distance of approximately 3 mm to the border of the sample. The spots and the head of the second mucosal swab smear were subsequently soaked in 2 ml NucliSens easyMAG (bio Merieux, Lyon, France) lysis buffer containing guanidiumisothiocyanate (GuSCN) and rocked at 150 rpm for 30 min at room temperature. The solution was then centrifuged at 1,500 g for 5 min. Afterwards the filter paper and the head of the smear were removed and 50 μL of silica particle solution (bio Merieux, Lyon, France) was added to each tube. Thereafter, the RNA extraction method originally described by Boom *et al*. was performed and then the nucleic acids were eluted from the silica with 30 μL elution buffer provided by the manufacturer [16].

For each extraction, a negative control of 100 μL of *Plasmodium*-negative full blood spotted on filter paper and a positive control of 100 μL of *Plasmodium*-spiked (NF54 strain) full blood spotted on filter paper was used. After extraction the samples were either stored at -80°C for a maximum of 24 hours or immediately amplified using NABSA method.

### Real-time quantitative nucleic acid sequence-based amplification (QT-NASBA)

The primer and probes for Pfs16-mRNA, Pfs25-mRNA and 18S-rRNA were chosen as described earlier and were used at a final concentration of 290 nM for the primers and 145 nM for the probes [17,18]. All oligonucleotides used in this study are summarized in Table 1.

**Table 1:** **Oligonucleotides used for NASBA, hybridization and *in vitro *** **RNA**

**Oligonucleotides**	**Sequence 5´ to 3´**
Pfs16.F WT	5‘-AGT TCT TCA GGT GCC TCT CTT CA-3‘
Pfs16.R WT	5’-T7-AGC TAG CTG AGT TTC TAA AGG GCA-3’
Pfs16.F Q	5’-CAA CAT GAA TAT TCG AAA GTT CAT ACC-3’
Pfs16.R Q	5’-AGA ATC ATC TCC TTC GTC TTC TTC-3’
Pfs16 probe	5’-6-FAM-CGATCG-GCT GTT GGA CCT AAT CTA ATC TAG GTG GA-CGATCG-Dabsyl-3‘
Pfs25.F WT	5‘-GAC TGT AAA TAA ACC ATG TGG AGA-3‘
Pfs25.R WT	5‘-T7-CAT TTA CCG TTA CCA CAA GTT A-3‘
Pfs25.F Q	5’-GAA TTC GAC TGT AAA TAA ACC ATG TGG AGA-3’
Pfs25.R Q	5’-AAG CTT CAT TTA CCG TTA CCA CAA GTT A-3’
Pfs25 probe	5‘-Texas Red-CGATCG-CCC GTT TCA TAC GCT TGT AA-CGATCG –Dabsyl-3’
18S.F WT	5’-GTC ATC TTT CGA GGT GAC TT-3’
18S.R WT	5’-AAC TTT CTC GCT TGC GCG AA-3’
18S probe	5’-6-FAM-CGATCG-GAG AAA TCA AAG TCT TTG GG-CGATCG-Dabsyl-3’
M13.F	5'-GTA AAA CGA CGG CCA GT-3'
M13.R	5'-CAG GAA ACA GCT ATG AC-3'

QT-NASBA was performed using the NucliSens Basic Kit for amplification according to the manufacturer’s manual at a final volume of 20 μL. Pfs16-mRNA, Pfs25-mRNA and 18S-rRNA were chosen as target sequences of 111 base pairs, 156 bp and 100 bp, respectively. Pfs16-mRNA is the earliest marker available to detect gametocytogenesis and is expressed in all sexual parasite stages. Pfs25-mRNA is expressed only in the female stage V gametocytes, which allows separate quantification of mature gametocytes [19,20]. 18S-rRNA is expressed in all sexual stages as well as asexual stages.

Amplification was performed using NucliSENS EasyQ® Basic Kit (bio Merieux, Lyon, France) according to the manufacturer’s manual and as described recently [9]. Positivity was measured with the time to positivity (TTP) according to the time at which the readings passed a given threshold.

### Artificial RNA of Pfs16 and Pfs25

The primers Pfs16.F Q and Pfs16.R Q were used to amplify a 481 bp region of encoding a domain located to the parasitophorous vacuole membrane as described by Schneider *et al*. [20]. The amplified DNA from the K1 culture was cloned into TOPO plasmid and transformed into TOP10 cells.

DNA was extracted from overnight cultures and served as the PCR template with M13 Primers. Subsequently, the required band was amplified and Gel purified with the QIAgen Gel Extraction Kit (Qiagen N V, Venlo, the Netherlands) and used for *in vitro* transcription with T7 RNA polymerase (AmpliScribe™ T7-Flash™ Transcription Kit by Epicentre® Biotechnologies, Madison, WI, USA).

To ensure the purity for large quantities of RNA, a DNA digestion and RNA purification (RNAeasy Minielute Cleanup Kit, by Qiagen N V, Venlo, the Netherlands) was performed. *In vitro* RNA was quantified by measuring the exact RNA amount and purity by Bio Analyzer (Agilent RNA 6000 Nano Kit, by Agilent Technologies, Santa Clara, CA, USA). A ten-fold dilution series from 1.28 * 105 to 1.28 *10^10^ per μL was used in each NASBA run as a standard normalization.

The primers Pfs25.F Q and Pfs25.R Q were used to amplify a 156 bp region of Pfs25 located in the osmiophilic bodies [20]. The *in vitro* RNA was obtained as described for Pfs16. A ten-fold dilution series from 9.06 * 105 to 9.06 * 10^10^ per μL was used in each NASBA run as standard. Lengths of RNA fragments after *in vitro* transcription were 623 bp and 298 bp for Pfs16 and Pfs25, respectively. The results of the Bio Analyzer run are presented in Additional file 1: Figure S1.

### Parasite culture

To gain RNA templates for artificial RNA and positive controls for the NASBA, parasites were obtained from a continuous *in vitro* parasite culture of *P. falciparum* (strain NF 54 and K1). The culture was grown in sterile filtered medium (RPMI 1640 supplemented with 25 mM HEPES, 25 mM sodium bicarbonate, 25 mg/l gentamicin sulphate, 2% D-glucose, and 10% human type AB serum, pH 7.4) with additional erythrocytes to uphold a haematocrit of 5%. New medium to feed the culture was added twice daily. The cultures were kept under a gas phase of 3% O_2_, 5% CO_2_ and 92% N_2_[21,22]. For gametocyte culture, medium without antibiotic was used. To gain gametocytes enriched cultures for positive controls, the cultures were treated with N-Acetyl-Glucosamine 5% from day 4 to day 9. Mature gametocytes were harvested at day 14 as described before [9].

### Sample size

A recent DNA-based malaria study showed a sensitivity of 73% and 32% for saliva and urine samples, respectively, compared to microscopy of blood [11], similar sensitivities were assumed for 18S-rRNA detection. The same applied for gametocyte-specific diagnostic although lower prevalences and sensitivities were assumed. Based on experience and literature [17,18], microscopic gametocytaemia in blood in 20% of the samples by reading 200 fields and in 40-60% by reading the whole slide were expected. Pfs16-mRNA and Pfs25-mRNA detection by QT-NASBA was assumed in 100% and in 70-90%, respectively. Choosing a confidence interval normal distribution value of 90% (z = 1.65), an accuracy of 0.1 and a prevalence of 90%, a sample size of at least 15 patients was calculated. Intended sensitivity was set 95%. Used formulae were: $\mathrm{TP}+\mathrm{FN}=z^{2}\times \frac{\left(\mathrm{SN}\left(1‒\mathrm{SN}\right)\right)}{W^{2}}\;\mathrm{and}\;N\left(\mathrm{sN}\right)=\frac{\mathrm{TP}+\mathrm{FN}}{P}$[23].

### Data analysis

Data were analysed with Microsoft Excel and Mathematica 9.0 (Wolfram Research, Champaign, IL, USA). The data from the NASBA runs, originally in Microsoft Excel format, were imported into the Mathematica programming environment. For Pfs16, for each of the six NASBA runs there was an RNA dilution series, which was used as a benchmark to calculate the RNA copy numbers for the patient samples. For each sample in each run, a time to positivity (TTP) was calculated by finding at what point the measurement went above a cut-off. In the case of Pfs16 this cut-off was 1.234306 fluorescence signal. The final results were not sensitive to the exact value of this cut-off because the normalization was made using the known RNA dilution series. Once the TTP for the RNA dilution samples had been calculated, a relationship between TTP and RNA copy numbers was calculated. This relationship was unique to each run due to the slight variations in experimental conditions, but the normalization of RNA copy numbers allows for this variation. After the relationship between TTP and RNA copy numbers had been calculated (a logarithmic relationship between 1/TTP and log_10 RNA copy number), the RNA quantities for the patient samples could be calculated.

Because there was a large variation in absolute RNA quantities between the patients, the absolute value of the RNA count was not a good comparison to make across the different quantification methods (venous blood, finger-prick blood, mucosa, etc), so instead the ratio between the venous blood measurements and the other measurements was calculated for each patient. The average for the ratios for each method was then calculated and the standard deviation in the ratios given. Thus, the final quantification for a given sample type was the average of the ratios of the RNA count between that method and the RNA count of venous blood. For example: where # patients = total number of patients.

A second measure was given for the power of a measurement technique. This was the total number of samples which came out as positive.

For Pfs25, the same procedure was used, but the cut-off for the TTP was calculated from the fluorescence mean of a set of negative controls (their value at 90 min) plus 20 standard deviations of these measurements. This ensured that no negative results would show up as false positives. In this case, the three negative NASBA measurements at 90 min were 1.00533, 1.00199 and 0.99369, giving a cut-off of 1.06196 mean fluorescence signal. Again, the final results were not sensitive to this value as the RNA dilution series normalization took into account the variation in cut-off to define the TTP.

For 18S the same procedure as the previous two was used, but there was no RNA quantification. Only the number of positive and negative samples using the different measurement techniques was quantified.

$$\begin{array}{l} \mathrm{Mean}\;\left(\mathrm{finger}\;\mathrm{prick}\;\mathrm{blood}\right)=\frac{1}{\#\;\mathrm{patients}}\\ \;\sum_{i=1}^{\#\;\mathrm{patients}}\frac{\mathrm{RNA}\;\mathrm{of}\;\mathrm{finger}\;\mathrm{prick}\;\mathrm{blood}\;\mathrm{for}\;\mathrm{patient}\;i}{\mathrm{RNA}\;\mathrm{of}\;\mathrm{venous}\;\mathrm{blood}\;\mathrm{for}\;\mathrm{patient}\;i} \end{array}$$

To calculate the RNA copy numbers, the molecular weight (MW) of the artificial RNA of Pfs16 and Pfs25 was calculated using the formula for single stranded RNA: MW = (An x 329.2) + (Un x 306.2) + (Cn x 305.2) + (Gn x 345.2) + 159. This resulted for Pfs16 in a MW of 200576.6 Dalton [Da] (MW = (211 x 329.2) + (174 x 306.2) + (112 x 305.2) + (126 x 345.2) + 159) and for Pfs25 in 96043.6 Da (MW = (90 x329.2) + (95 x 306.2) + (46 x 305.2) + (67 x 345.2) + 159). RNA/μL was given in ng/μL by the Bio Analyzer. The amount of RNA/μL of the measured peaks which represented either Pfs16 or Pfs25 was divided by their MW to gain the copy number/μL: Pfs16 = 2.5600867417625E + 15 [Da/μL]/ 200576.6 Da = 12 763 636 146/μL and Pfs25 = 8.7042949219927E + 15 Dalton [Da/μL]/ 96043.6 Da = 90 628 578 291/μL).

## Results

### Baseline data

The study centre is situated in the hilly area near Jimma at around 1,650 m above sea level. The study was implemented during the routine diagnostic practices of the clinic. Sixteen patients with microscopically uncomplicated *P. falciparum* malaria were enrolled. Ninety-six samples from 16 patients were extracted and subsequently analysed by QT-NASBA. One patient was negative for all targets, species PCR revealed infection with *Plasmodium malariae*; the patient was therefore excluded from the study.

The median age of the 15 remaining patients was 18 years (range 14-70 years); 33% were female and 40% had fever at admission. The median temperature at admission was 37.0°C (range 36.4-40.0°C). The geometric mean of the parasitaemia was calculated to be 9,320/μL (95% CI 7,298-11,701/μL). No gametocytes could be detected during initial microscopy of 200 fields at recruitment. Repeated microscopic examination of the whole blood slide revealed a median gametocyte density of 7 stage V gametocytes per 10 μL (range 3-29) in the slides of 10 patients. In five patients, none were detectable in 10 μL.

### Qualitative RNA detection in patient material

**Figure 1:**
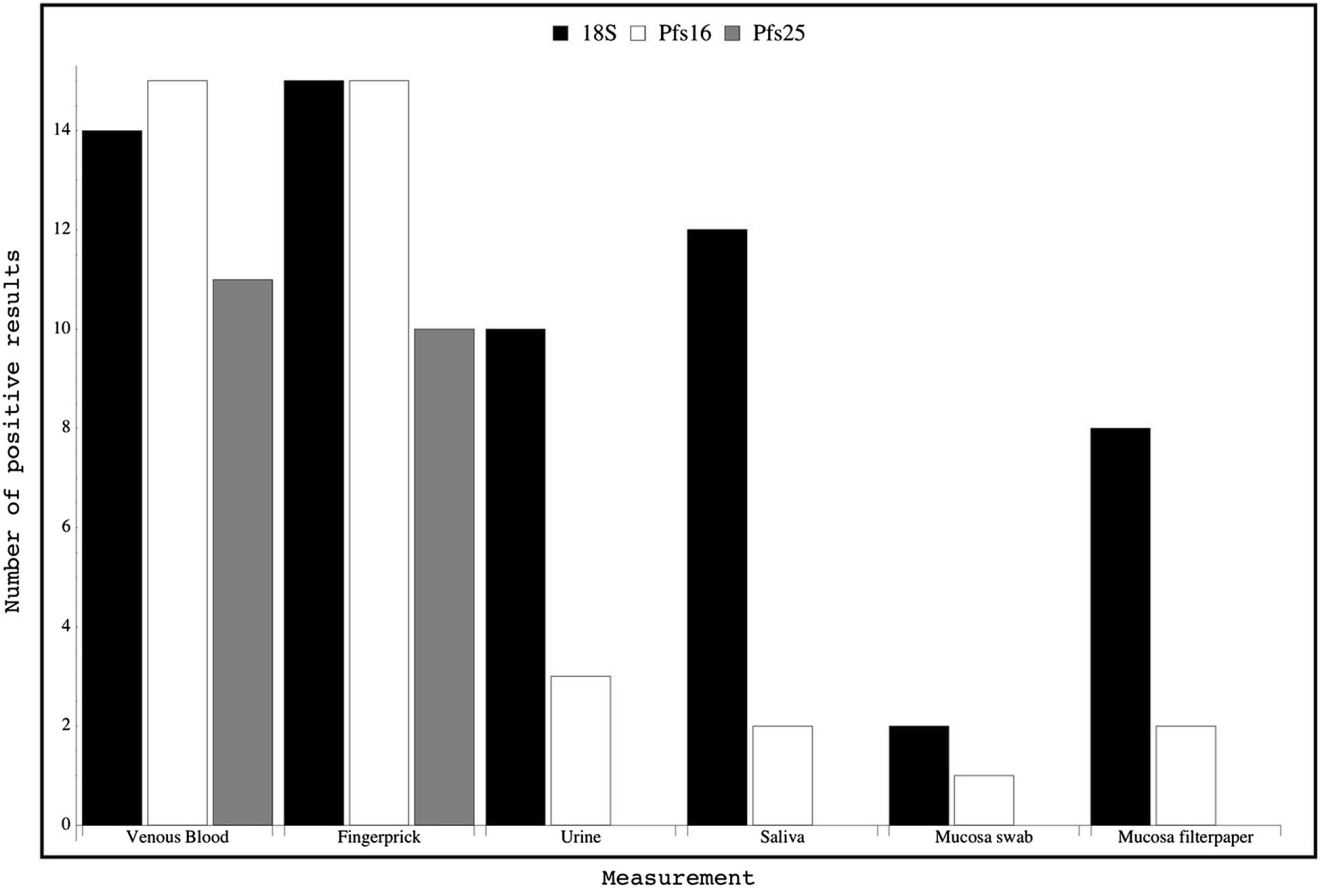
**Qualitative data for 18S-rRNA, the marker for asexual and sexual parasites, Pfs16-mRNA, the marker for all sexual stages, and Pfs25-mRNA, the marker for mature sexual stages (n = 15).** 18S-rRNA: Sensitivity for saliva and urine were 80% and 67%, respectively. Detection in mucosa samples was the least sensitive method. Pfs16-mRNA: Detection of the target in non-invasively collected material was low. Urine performed best with a sensitivity of 20%. Pfs25-mRNA: Detection of mRNA amplicons was only achieved in blood samples. Sensitivity of the methods or size of the RNA fragments in other compartments seems to low for detection with this method.

Table 2 shows qualitative data for RNA detection of all three targets. The marker for asexual and sexual parasites, 18S-rRNA, was detectable in all finger-prick samples (Figure 1). Venous blood samples from one patient remained repeatedly negative although geometric mean parasitaemia showed an average density level when compared with the other patients. Mutations at the primer binding site or inhibition were unlikely as finger-prick blood was positive.

**Table 2:** Qualitative QT-NASBA results for Pfs16, Pfs25 and 18S in different patient material

	**Pfs16**	**Pfs25**	**18S**
	**m/N (%)**	**m/N (%)**	**m/N (%)**
Venous blood 100 μL	15/15 (100.0)	11/15 (73.3)	14/15 (93.3)
Finger prick 25 μL	15/15 (100.0)	10/15 (66.7)	15/15 (100.0)
Urine 100 μL	3/15 (20.0)	0	10/15 (66.7)
Saliva 100 μL	2/15 (13.3)	0	12/15 (80.0)
Mucosa swab	1/15 (6.7)	0	2/15 (13.3)
Mucosa filter paper	2/15 (13.3)	0	8/15 (53.3)

(m = positive samples, N = all samples).

18S-rRNA detection in saliva was sensitive (80%) but the target could only be amplified in two-thirds and in one-half of the urine and mucosa samples, respectively. Interestingly, the mucosa on filter paper performed four times better than the mucosa swab preparation.

RNA amplification with Pfs16 primers showed 100% sensitivity in blood samples. Detection of Pfs16-mRNA in saliva, urine and mucosa was low (Figure 1). Urine performed best of these with a detection rate of 20%.

RNA from mature gametocytes was detectable in venous blood samples from 11 patients and in finger-prick samples from ten patients. The blood slide from the discordant pair showed no gametocytes in the microscopic investigation. The non-invasive material was completely negative for mature gametocytes by QT-NASBA (Figure 1).

### Quantitative RNA detection in patient material

**Figure 2:**
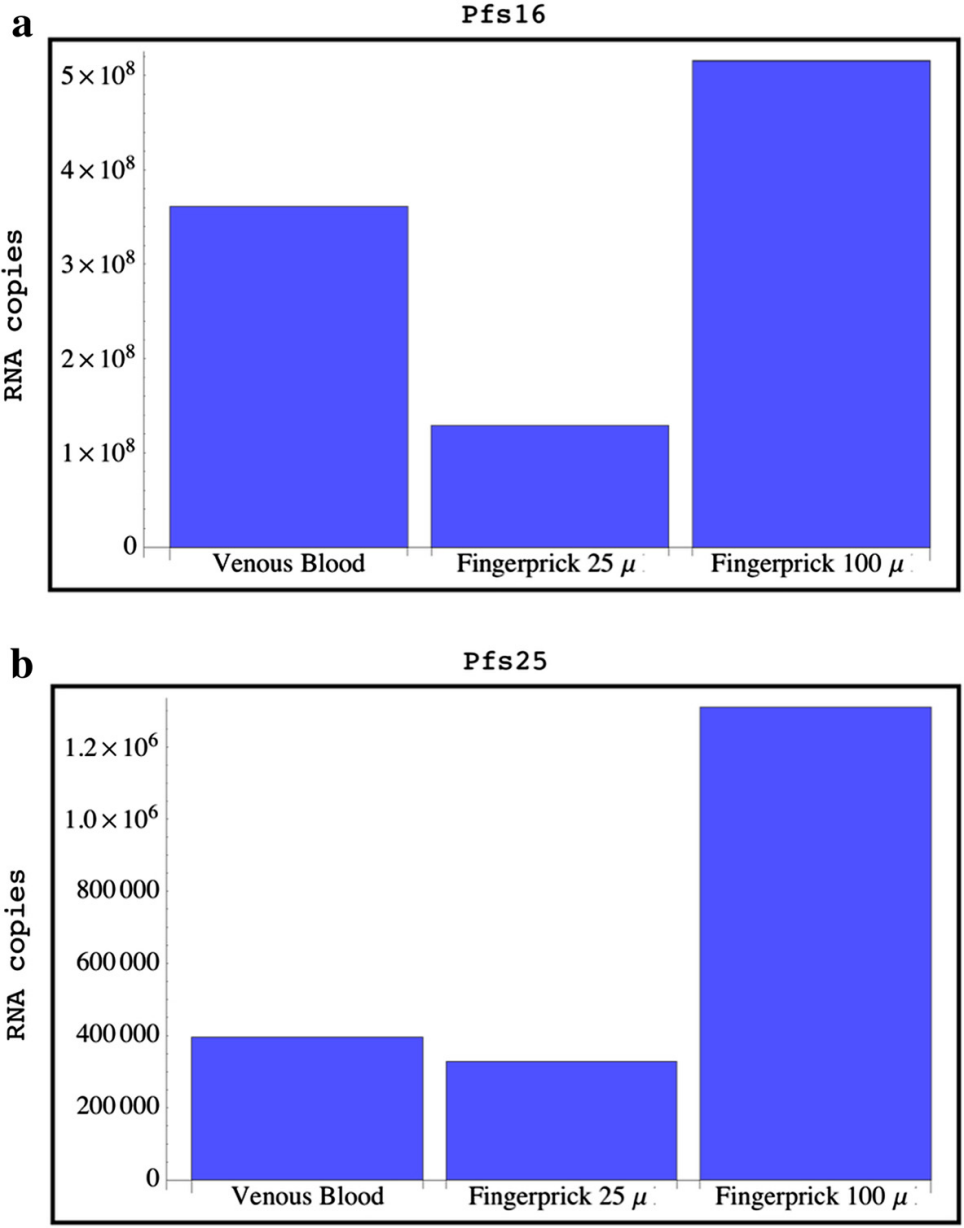
**Quantitative data for Pfs16-mRNA (a), the marker for all sexual stages, and for Pfs25-mRNA (b), the marker for mature sexual stages (n = 15).** The amount of RNA copy numbers in 25 μL of finger-prick blood was compared to 100 μL of venous blood. The difference was not significant. The theoretical amount in 100 μL of finger-prick blood was presented to underline the different amounts in capillary and venous blood. The difference was more prominent for Pfs25-mRNA expressed in mature gametocytes than for Pfs16. 3.3 times more copy numbers could be assumed in 100 μL capillary blood compared to venous blood.

The limit of detection was 143 RNA copy numbers for Pfs16. Quantification of Pfs16 copy numbers showed 1.43 times more RNA copies in finger-prick blood compared to venous blood (Figure 2a). Twenty-five μL of finger-prick blood would correspond to 35.75 μL of venous blood.

As the sample size was very small and the distribution of RNA quantities was unknown, parametric significance tests on the results were inadequate. Therefore sign significance tests were chosen. For Pfs16, 11 of the 15 patients had higher RNA readings for theoretical 100 μL of finger-prick blood than 100 μL of venous blood. However, the difference was not significant (p-value 0.095) by Wilcoxon Signed Rank test. On the other hand, the null hypothesis that data from 25 μL of finger-prick blood and 100 μL venous blood were the same could not be rejected (p-value 0.12) by Wilcoxon Signed Rank test.

The limit of detection for Pfs25 was 1,710 RNA copy numbers. Quantification of Pfs25 copy numbers showed 3.3 times more RNA copies in theoretical 100 μL finger-prick blood compared to venous blood (Figure 2b). Twenty-five μL of finger-prick blood would correspond to 82.6 μL of venous blood.

Four samples were negative with both methods, eight of the remaining 11 patients had higher RNA readings for 100 μL of finger-prick blood than 100 μL of venous blood. The null hypothesis that data from 100 μL of finger-prick blood and 100 μL venous blood were the same could be rejected (p-value <0.014) by Wilcoxon Signed Rank test. Comparing 100 μL venous blood and 25 μL finger-prick blood, there was no evidence for a real difference. The null hypothesis that data from 25 μL of finger-prick blood and 100 μL venous blood were the same could not be rejected (p-value 0.964) by Wilcoxon Signed Rank test.

## Discussion

In this study, 90 samples from 15 microscopically positive *P. falciparum* patients from southwest Ethiopia were tested for falciparum-specific RNA by QT-NASBA. Microscopy showed mild to moderate parasitaemia in all, and low gametocytaemia in 80% of patients. Median gametocyte density was 7 per 10 μL (range 3-29) corresponding to a density of 0.3-2.9 gametocytes/μL which is well below the usual threshold for microscopy of at least 16 gametocytes per μl [24]. Gametocytaemia was only detected by reading the complete thick smear of 10 μL blood, no gametocytes were detected by routinely reading 200 fields.

The limit of detection for the gametocyte-specific target Pfs16 was 143 copy numbers corresponding to very low submicroscopic densities of gametocytes.

All blood samples were positive for Pfs16-mRNA that is expressed in all gametocyte stages. Only stage I, microscopically indistinguishable from asexual stages, and stage V, the mature gametocytes, are found in peripheral blood in humans. Stages II to IV are sequestered away from circulation [25]. Pfs25-mRNA from mature gametocytes was detectable in 11 patients. The most probable explanation for this is that the other four patients had no mature gametocytes in their peripheral blood yet. Diagnosis of the disease is most often shortly after onset of symptoms, mature stages peak at day 7 after onset of symptoms [25-27], although this is dependent on the treatment. Secondly, the theoretical limit of detection for Pfs25 was 1,710 RNA copy numbers, about ten times less sensitive than Pfs16. However, stage-specific expression levels per gametocyte are not published for Pfs25 or Pfs16, average concentration levels of the specific targets are unknown.

As expected, quantification of copy numbers showed that the density of RNA copy numbers in capillary blood (finger prick) was higher than in blood from the cubital vein [28]. The difference was more prominent for mature gametocytes than for stage I and V together. The chance to be taken up by mosquitoes is higher in small blood vessels and more important for stage V.

Comparison of 25 μL finger prick and 100 μL venous blood showed a higher sensitivity for venous blood but the difference for Pfs25 was negligible. However, Pfs25 was not detectable in a finger-prick sample from one patient where the venous blood sample was positive. Although the density in capillary blood seems to be higher, the amount of blood extracted was only 25 μL. For venous blood, 100 μL blood was extracted. Naturally, the probability to detect any gametocyte increases with the amount of blood extracted if density is very low. As finger-prick blood is more convenient and simpler to get in rural areas with its associated health centres, 25 μL finger-prick blood can be recommended for mature gametocyte screening in epidemiological settings.

Probably all or most of the patients with asexual parasitaemia carry gametocytes but sensitive rapid diagnostic and subsequent elimination of all parasites is essential in symptomatic malaria. However, as transmission reduction is of key importance in eradication efforts, gametocyte-specific diagnostic is needed to control the success of interventions. Gametocyte carriage is age-dependent and decreases with age [25,29]. Density of gametocytes influences the infection of mosquitoes, very low densities are sufficient but the relation seems non-linear [24]. Investigation of the infectious reservoir includes testing for asymptomatic carriers. This would have been the next step of this study if non-invasive methods had proved more sensitive. Symptomatic malaria patients were chosen for the pilot project as higher gametocyte densities were assumed. To investigate further and reduce the infectious reservoir, finger prick blood spotted on filter paper seems to be a reliable field-proven method but should be validated for asymptomatic carriers.

Gametocyte-specific mRNA detection in saliva, urine and mucosa was low for Pfs16 and negative for Pfs25. Either mature gametocyte mRNA is not secreted at all or the sensitivity of the method is too low. Pfs16 could be found in 20% of urine and 13% of saliva and mucosa samples. Therefore, the detection rate for non-invasive methods is unfortunately insufficient. With the methods used, the fragments amplified were 111 bp for Pfs16-mRNA and 156 bp for Pfs25-mRNA, respectively. It is known that mRNA is fragile and decomposes fast outside of cells. The milieu found during urine filtration and within urine, as well as on mucosal surfaces is especially delicate for extracellular mRNA stability. Therefore, part of the poor detection sensitivity may be due to the decay of the mRNA into smaller pieces in the respective compartment. Under the given circumstances, finger-prick blood collection proved to be the most effective method. Filter paper could be stored at room temperature if it is processed within 28 days. RNA loss would be on average about 20%. If storage duration is longer than one month, RNA loss at room temperature is unacceptably high and the filter paper should be frozen [9].

Interestingly, mucosa on filter paper performed two to four times better than fresh frozen mucosal swab. The swab seems to conserve the RNA less inefficiently than instant drying and freezing of mucosal swab material on dry filter paper.

A study from The Gambia on DNA of asexual parasites showed a good correlation of saliva parasite density as estimated by quantitative real-time PCR (qPCR) with parasite counts established by microscopy (p = 0.58; P <0.001). Correlation of qPCR results for urine with microscopy counts was poor (p = 0.20; P =0.117). Mean parasite density in blood was about 600-fold and 2,500-fold greater than mean density in saliva and urine samples, respectively. Compared with microscopy results, nested PCR of saliva had a sensitivity of 73% for all samples and a sensitivity of 82% in samples with a parasite density of > or = 1,000 parasites/μL; sensitivity in urine samples was only 32% [11]. PCR-amplicons had a bigger size than amplicons of the NASBA, sensitivity was nevertheless similar. The advantage of RNA is that its detection indicates that there are still living parasites circulating in the blood whereas DNA can be positive several days after acute infection.

The study from Mharakurwa et *al*. compared blood, saliva and urine samples from individuals with mostly aymptomatic parasitaemia. Samples were spotted on filter paper before extraction. Saliva samples showed a 1.6-fold higher amplification score than corresponding urine samples (p = 0.029). DNA amplicons ranged between 229 bp and 643 bp [10].

In this study, 18S-rRNA in saliva was detected in 80% of the patients. 67% of urine samples yielded RNA. However, geometric mean parasitaemia (GMP) by microscopy was higher than in the other studies. GMP in The Gambia was 1,785 parasites/μL (CI 95% 695–4,588 parasites/μL) [11]. Furthermore, samples in the Gambian study were processed directly within two hours and not spotted on filter paper before. A recent study about filter paper showed better results for mRNA detection when the blood was spotted on filter paper and dried 24 hours before extraction than direct extraction of fresh blood [9].

Urine and mucosa tissue proved inefficient for the detection of malaria RNA but saliva analysis as non-invasive method could be conducted in cross-sectional and other studies. Primers amplifying smaller fragments of the target RNA may increase the sensitivity of the test protocol. Saliva could be tested with various diseases including malaria. Again, a rapid test would simplify efforts and allow immediate treatment.

## Conclusion

Non-invasively collected material such as urine, saliva or mucosal swabs seemed unsuitable for the detection of gametocyte-specific mRNA when protocols established for blood are used. Detection of 18S-rRNA in saliva was achieved in 80% of the patients. Combined screening methods for malaria and other diseases such as tuberculosis with saliva sampling might speed up diagnosis in future. As suspected, finger-prick testing revealed the highest absolute count of RNA copies per μL, especially for Pfs25-mRNA copies. Comparing the two methods of taking 25 μL finger-prick blood and drawing 100 μL venous blood for Pfs25-mRNA, 17.4% less RNA copies in the finger-prick blood seem acceptable in rural health centres, considering the ease of withdrawal, the low costs and the tiny blood volume - especially in small children - by finger-prick test. The method should be recommended for quantification of gametocyte concentrations.

## Competing interests

The authors declare that they have no competing interests.

## Authors’ contributions

KK collected and analysed the samples, produced the dilution series and was involved in drafting of the manuscript. NBR designed the study, took care of the *Plasmodium* cultures, coordinated the laboratory procedures and drafted the manuscript. AZ coordinated the study in Jimma and was involved in manuscript preparation. NA was involved in sample collection and coordination. TE was involved in coordination and design of the study. TL was involved in design and preparation of the manuscript. AW was involved in statistics and manuscript preparation. JS was responsible for data analysis, statistics and manuscript design. MP coordinated the study and helped drafting the manuscript. All authors read and approved the final manuscript.

## Supplementary Material

Additional file 1 **Bio Analyzer results.** The Bio Analyzer run shows the purity of RNA copies for Pfs16 and Pfs25 presented each by a specific peak. Click here for file
