# Supplementary figures and images for: Evaluation of Plasmodium falciparum gametocyte detection in different patient material
Source: Malar J. 2013 Dec 4;12:438. doi: 10.1186/1475-2875-12-438 (PMC4234195; doi:10.1186/1475-2875-12-438)

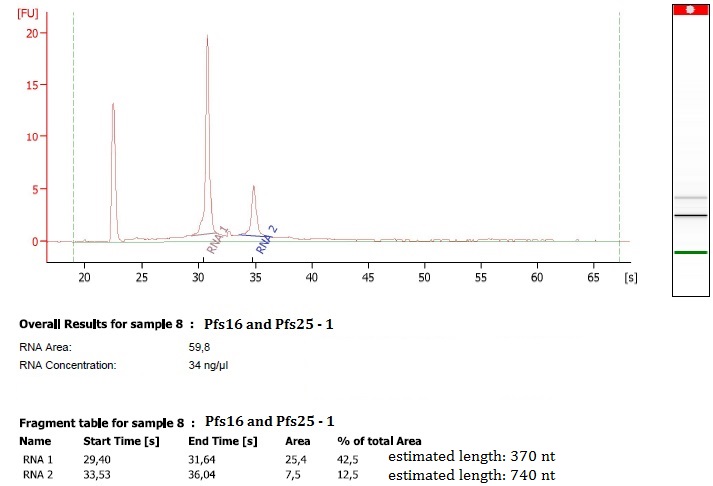

Supplement: Additional file 1 — Bio Analyzer results. The Bio Analyzer run shows the purity of RNA copies for Pfs16 and Pfs25 presented each by a specific peak. [file 1475-2875-12-438-S1.jpg]
